# Transcriptional and Epigenetic Factors Associated with Early Thrombosis of Femoral Artery Involved in Arteriovenous Fistula

**DOI:** 10.3390/proteomes10020014

**Published:** 2022-04-30

**Authors:** Vikrant Rai, Devendra K. Agrawal

**Affiliations:** Department of Translational Research, Western University of Health Sciences, Pomona, CA 91766, USA; vrai@westernu.edu

**Keywords:** arteriovenous fistula, early thrombosis, epigenetic regulation, maturation failure, microRNAs, transcription factors, transcriptional regulation

## Abstract

Arteriovenous fistulas (AVFs), created for hemodialysis in end-stage renal disease patients, mature through the outward remodeling of the outflow vein. However, early thrombosis and chronic inflammation are detrimental to the process of AVF maturation and precipitate AVF maturation failure. For the successful remodeling of the outflow vein, blood flow through the fistula is essential, but early arterial thrombosis attenuates this blood flow, and the vessels become thrombosed and stenosed, leading to AVF failure. The altered expression of various proteins involved in maintaining vessel patency or thrombosis is regulated by genes of which the expression is regulated by transcription factors and microRNAs. In this study, using thrombosed and stenosed arteries following AVF creation, we delineated transcription factors and microRNAs associated with differentially expressed genes in bulk RNA sequencing data using upstream and causal network analysis. We observed changes in many transcription factors and microRNAs that are involved in angiogenesis; vascular smooth muscle cell proliferation, migration, and phenotypic changes; endothelial cell function; hypoxia; oxidative stress; vessel remodeling; immune responses; and inflammation. These factors and microRNAs play a critical role in the underlying molecular mechanisms in AVF maturation. We also observed epigenetic factors involved in gene regulation associated with these molecular mechanisms. The results of this study indicate the importance of investigating the transcriptional and epigenetic regulation of AVF maturation and maturation failure and targeting factors precipitating early thrombosis and stenosis.

## 1. Introduction

Arteriovenous fistulas (AVFs) provide vascular access for hemodialysis in patients with end-stage renal disease [1]. The successful use of an AVF for hemodialysis depends on the maturation of the fistula through the outward remodeling of the outflow vein to make it appropriate to respond to increased blood flow so that it can be repeatedly cannulated to provide adequate flow for dialysis [2,3]. The maturation rates of AVFs have been reported to range between 40% and 80%, but these rates decrease with aging, a distal fistula location, and a small vein diameter [4]. Non-maturation of AVF due to early thrombosis, chronic inflammation, and the failure of outward remodeling is an important cause of AVF failure. Early thrombosis can occur due to hematoma formation, a hypercoagulation state, decreased blood flow rates, intimal injury during AVF creation, and endothelial cell dysfunction, leading to chronic inflammation and the failure of outward remodeling [2]. Along with thrombosis, stenosis and neointimal hyperplasia (NIH) of the outflow vein also contribute to early AVF failure [5,6]. Chronic inflammation involving various mediators of inflammation, including triggering receptor expressed on myeloid cells-1 (TREM-1) and toll-like receptor 4 (TLR4), plays a crucial role in NIH, thrombosis, and stenosis [7,8,9,10,11]. To attenuate vessel stenosis after AVF creation, we targeted chronic inflammation by inhibiting TREM-1 using LR-12 peptides and TLR4 with TAK-242 and performed sequencing in collected tissues to investigate the changes in gene expression associated with stenosis and thrombosis. Recently, we reported on differentially expressed genes (DEGs) associated with stenosis and thrombosis of the femoral artery involved in AVF [12].

The presence of differentially expressed genes in the thrombosed artery that are involved in AVF indicates the change in gene expression associated with AVF creation and failure due to thrombosis. Since the expression of genes is regulated at both transcriptional and post-transcriptional levels [13], we investigated the transcription factors and microRNAs associated with either activated or inhibited networks and regulating the expression of various DEGs expressed in our data set (hereafter referred to as activated or inhibited).

Transcription factors (TFs) are regulatory proteins involved in the process of converting DNA to RNA (transcription) that allow the unique expression of a gene in different cell types. The function of TFs is to activate the transcription of DNA but TFs rarely inhibit gene expression [13]. MicroRNAs (miRs) belong to the class of small non-coding RNAs (18- to 25 nucleotide long), which are involved in controlling gene expression post-transcriptionally by targeting mRNAs based on sequence complementarity [14,15]. An miRNA binds with its target messenger RNA (mRNA) and blocks its translation or promotes its degradation, thereby decreasing gene expression. However, in opposition to the consensus that miRNA reduces gene expression, there is evidence that some miRNAs can upregulate gene expression [16,17]. Furthermore, TFs, including nuclear factor kappa beta (NF-κB), PU.1, the Ets-1 family, activator protein 1 (AP1), Krϋppel-like factor 2 (KLF2), zinc fingers and homeoboxes 2 (Zhx2), and activating transcription factor 4 (ATF4), play a critical role in atherosclerosis [18]. Since TFs and miRNAs regulate gene expression, targeting TFs and miRNAs to modulate gene expression favoring attenuated thrombosis, stenosis, and plaque formation will be of therapeutic importance. Decreased thrombosis, stenosis, and excessive NIH will render arteries and veins patent, and this may increase AVF patency. In this study, we have revealed various novel TFs and miRNAs associated with early thrombosis of the femoral artery involved in AVF and regulating various differentially expressed genes (DEGs) associated with early thrombosis. Thus, targeting these TFs and miRNAs might be of therapeutic and clinical significance.

## 2. Materials and Methods

The material and methods involved in creating AVFs, assessing pre-and post-operative femoral vessels involved in AVFs, tissue harvesting, tissue processing, histology, immunostaining, and bulk RNA sequencing have been described previously [12]. We choose the swine model because the swine model represents the best choice for studies of occlusive arterial and venous diseases and there are similarities in the anatomy, physiology and pathological responses of the human and porcine cardiovascular systems [19]. Additionally, this approach enables us to use the same catheters and tools as those used clinically in humans. Female swine were chosen because female swine are easy to handle compared to male swine and because of the fact that, due to their aggressive behavior, nearly 100% of male pigs have been castrated chemically or immunologically, and such procedures change the hormonal pattern and pathophysiological responses that could significantly affect the outcomes and results due to their effect on immune and resident cells. Briefly, following the creation of AVFs in Yucatan miniswine, LR12 (TREM-1 antagonist) + TAK242 (TLR4 antagonist) were locally administered at the site of fistula creation. For controls, a mixture of scrambled peptide +30% ethanol as the vehicle of TAK-242 was used. LR12 was injected once during the surgery and >10^9^ lentiviral particles in 1 mL were injected. TAK-242 was administered at a dose of 3 mg/kg as bolus and then 0.1 mg/kg daily for 7 days and then once weekly for 4 weeks as a maintenance dose. The patency of the femoral artery and vein was assessed with doppler ultrasound, angiography, and optical coherence tomography at the end of 12 weeks and the swine were sacrificed for the harvesting of tissues. Femoral arteries from the AVF site and from the contralateral sides were collected. The total RNA, isolated using the TRIzol method from three thrombosed femoral arteries and three contralateral femoral arteries, was sent for bulk RNA sequencing. RNA samples with RIN > 6 were subjected to library preparation and sequencing using the NEBNext Ultra II RNA Library Prep Kit for Illumina and Illumina HiSeq instruments at Genewiz LLC (Plainfield, NJ, USA) as mentioned in [12]. IPA was used for the analyses of microRNAs and transcription factors, as detailed in the following sections.

### 2.1. Ingenuity Pathway Analysis

In order to perform the functional analysis to investigate the novel transcription factors and microRNAs, ingenuity pathway analysis (IPA) was conducted by uploading the identified genes from bulk RNA sequencing. The functional IPA analysis and the statistical inference was completed at Bioinformatics and Systems Biology Core at the University of Nebraska Medical Center (UNMC, Omaha, NE, USA). IPA pathway analyses, including causal network and regulator effect analyses, were performed using IPA (QIAGEN Inc., Hilden, Germany, https://digitalinsights.qiagen.com/products-overview/discovery-insights-portfolio/analysis-and-visualization/qiagen-ipa/ (accessed on 31 August 2021)). Causal network analysis was conducted to examine causal relationships associated with input genes by expanding the upstream analysis to include regulators that are not directly connected to targets in the dataset, as well as to identify potential therapeutic or toxicity targets and known drugs and biomarkers. The analysis of regulator effects provides unprecedented insights into the input data by integrating upstream regulator results with the results of the downstream effects to create causal hypotheses. The results of regulator effects analysis explain the way in which the events occurring upstream cause a particular phenotypic or functional outcome downstream (https://digitalinsights.qiagen.com (accessed on 31 August 2021)).

### 2.2. Search Tool for the Retrieval of Interacting Genes/Proteins (STRING) Network Analysis

Transcription factors regulate the expression of a gene, which in turn regulates protein expression through translation. To delineate the protein–protein interactions regulated by the TFs that appeared in our data set, we performed STRING network analysis using https://string-db.org/ (accessed on 19 February 2022).

## 3. Results

IPA analysis revealed microRNAs associated with activated and inhibited networks. The upstream regulator analysis using input genes from the LR12 + TAK242-treated group compared to the contralateral femoral arteries revealed 15 microRNAs (mir-193, mir-365, miR-199a-5p, miR-889-3p, mir-889, mir-1, mir-137, miR-1195, mir-644, miR-293-5p, mir-122, miR-224-5p, mir-153, mir-346, and mir-467; mir-microRNA, and miR-mature microRNA) but none were associated with activated or inhibited networks. The causal network analysis using input genes from the LR12 + TAK242-treated group compared to the contralateral femoral arteries revealed nine microRNAs (miR-889-3p, mir-889, mir-365, miR-199a-5p, mir-15, mir-379, miR-23a-3p, mir-193, miR-96-5p; mir-microRNA, and miR-mature microRNA) but these were not associated with activated or inhibited networks. The upstream regulator analysis using input genes from the LR12 + TAK242-treated group compared to the scrambled peptide + ethanol group arteries revealed 35 microRNAs, and of these, three were inhibited (Table 1) and 32 microRNAs (mir690, miR-1258, miR-690, mir-1258, mir-129, mir-219, mir-363, mir-584, mir-132, miR-150-5p, miR-375-3p, mir-142, mir-204, miR-30c-5p, miR-204-5p, miR-4728, miR-101b-3p, miR-185-3p, miR-4728-3p, mir-331, mir-663, mir-210, mir-451, mir-33, miR-138-5p, miR-483-3p, mir-155, mir-8, mir-30, mir-17, mir-223, and miR4269; mir-microRNA, and miR-mature microRNA) were not associated with activated or inhibited networks. The causal network analysis, using input genes from the LR12 + TAK242-treated group compared to scrambled peptide + ethanol group arteries, revealed 17 microRNAs and, of these, one microRNA was associated with an activated network and four microRNAs were associated with inhibited networks (Table 2), whereas 12 microRNAs (mir-124, mir-326, mir-181, mir-132, miR-4269, miR-138-5p, miR-1258, miR-690, mir-1258, mir-219, mir-584, miR-690; mir-microRNA, and miR-mature microRNA) were not associated with activated or inhibited networks. The identified microRNAs were associated with various differentially expressed genes (DEGs) with log_2_ > 2 or <−2 with *p* < 0.05 and other DEGs with log_2_ < 2 or >−2, *p* < 0.05. From the list of all DEGs, we sorted out the DEGs involved in NIH, plaque formation, endothelial cell (EC) dysfunction, phenotypic changes in VSMCs, proliferation and migration of ECs and VSMCs, angiogenesis, vasculogenesis, immune cell recruitment, and inflammation based on an extensive literature search (the complete list of microRNAs can be found in Supplementary File S1).

IPA analysis revealed transcription factors associated with activated and inhibited networks. The upstream regulator analysis using input genes from the LR12 + TAK242-treated group compared to contralateral femoral arteries revealed 132 TFs (Supplementary File S2 sheet 1). Among 132 TFs, 11 TFs were associated with activated networks, whereas four TFs were associated with inhibited networks (Table 3). The causal network analysis using input genes from the LR12 + TAK242-treated group compared to contralateral femoral arteries revealed 91 TFs (Supplementary File S2 sheet 2) and of these, 13 were associated with activated and 14 were associated with inhibited networks (Table 4). The upstream regulator analysis using input genes from the LR12 + TAK242-treated group compared to scrambled peptide + ethanol group arteries revealed 311 TFs (Supplementary File S2 sheet 3) and of them, 14 were associated with activated and 11 TFs were associated with inhibited networks (Table 5). The causal network analysis using input genes from the LR12 + TAK242-treated group compared to scrambled peptide + ethanol group arteries revealed 152 TFs (Supplementary File S2 sheet 3) and of these, 22 TFs were associated with activated and 25 TFs were associated with inhibited networks (Table 6). The identified TFs were associated with various differentially expressed genes (DEGs) with log_2_ > 2 or <−2 with *p* < 0.05 and other DEGs with log_2_ < 2 or >−2, *p* < 0.05. From the list of all DEGs, we sorted out the DEGs involved in NIH, plaque formation, endothelial cell (EC) dysfunction, phenotypic changes in VSMCs, proliferation and migration of ECs and VSMCs, angiogenesis, vasculogenesis, immune cell recruitment, and inflammation based on an extensive literature search (the complete list of TFs can be found in Supplementary File S2).

**Figure 1:**
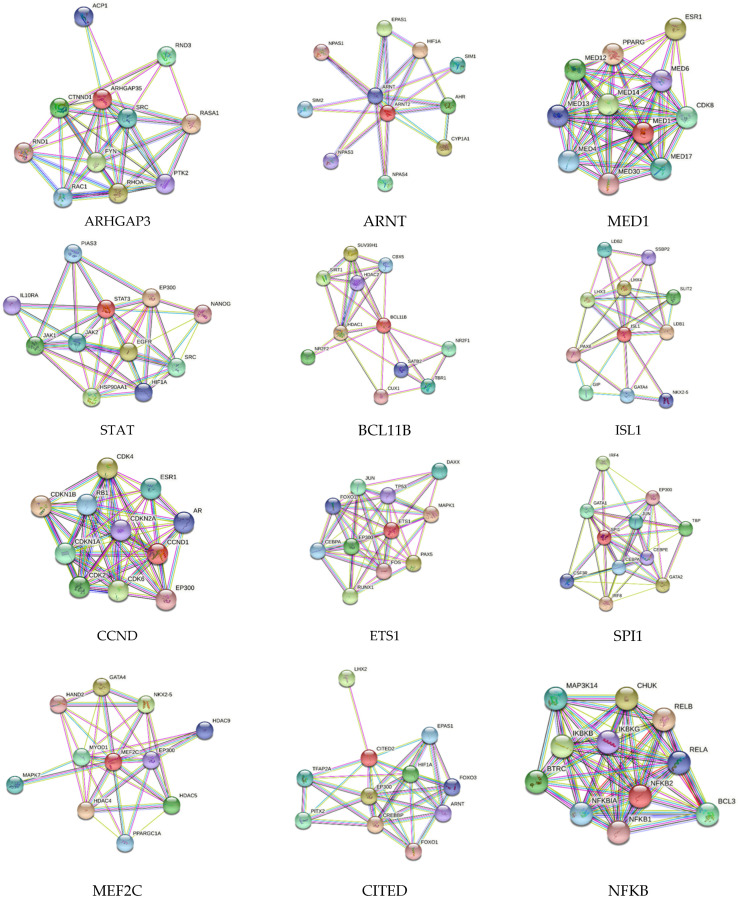
STRING network analysis for protein–protein interactions for Rho GTPase-activating protein 35 (ARHGAP35), aryl hydrocarbon receptor nuclear translocator 2 (ARNT2), mediator of RNA polymerase II transcription subunit 1 (MED1), signal transducer and activator of transcription 3 (STAT3), B-cell lymphoma/leukemia 11B (BCL11B), insulin gene enhancer protein ISL-1 (ISL1), G1/S-specific cyclin-D1 (CCND1), transcription factor PU.1 (SPI1), myocyte-specific enhancer factor 2C (MEF2C), Cbp/p300-interacting transactivator 2 (CITED2), and nuclear factor NF-kappa-B p100 subunit (NFKB2).

**Figure 2:**
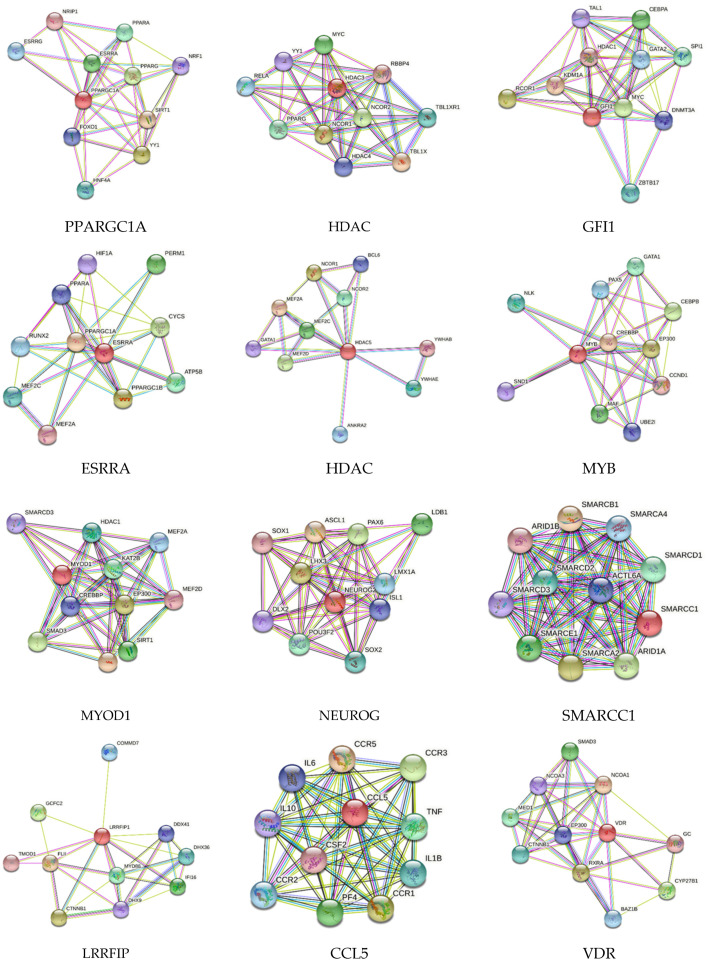
STRING network analysis of protein–protein interaction for peroxisome proliferator-activated receptor gamma coactivator 1-alpha (PPARGC1A); histone deacetylase 3 (HDAC3); zinc finger protein Gfi-1 (GFI1); steroid hormone receptor ERR1 (ESRRA); histone deacetylase 5 (HDAC5); transcriptional activator Myb (MYB); myoblast determination protein 1 (MYOD1); neurogenin-2 (NEUROG2); Swi/snf-related, matrix-associated, actin-dependent regulator of chromatin subfamily c member 1 (SMARCC1); leucine-rich repeat flightless-interacting protein 1 (LRRFIP1); C-C motif chemokine ligand 5 (CCL5); and vitamin D receptor (VDR).

STRING network analysis showed the interaction of various proteins with each other. STRING network analysis for genes (encoded by genes being regulated by TFs in this study) showed protein–protein interactions among each other. Specifically, ARNT2 showed interaction with HIF-1α; BCL11B with SIRT2, HDAC1, and HDAC2; CITED2 with HIF-1α, ARNT, FOXO1, and CREBBP; ETS1 with FOXO1, JUN, MAPK1, and FOS; MED1 with PPARG and ESR1; MYOD1 with HDAC4, PPARGC1A, HDAC5, MEF2C, and GATA4; NFKB2 with NFKB1, RELA, and RELB; SPI1 with GATA1, CEBPA, and JUN; and STAT3 with JAK1, JAK2, HIF-1α, IL10RA, and HSP90AA1 (Figure 1). Similarly, CCL5 interacted with CCR2, CCR3, CCR5, CCR1, IL6, IL10, IL1B, and TNF; RSRRA with PPARGC1A, HIF-1α, and MEF2C; GFI1 with SPI1; HDAC3 with RELA, PPARG, and HDAC4; HDAC5 with MEF2A; NEUROG2 with ISL1; and VDR with MED1 (Figure 2). The analysis showed protein–protein interaction by the proteins involved in vascular pathologies, immune cell responses, inflammation, and hypoxia and this suggests their probable role in AVF maturation and maturation failure.

## 4. Discussion

Intimal injury during AVF creation induces NIH, which helps in vascular remodeling and makes vessel suitable for increased blood flow through the outflow vein. However, excessive NIH and early thrombosis of the inflow artery or excessive NIH in the outflow vein can lead to vessel stenosis and AVF maturation failure [12]. Neointimal reendothelialization, with the aim of obtaining an intact endothelium, is necessary for the proper healing and AVF maturation and involves endothelial cell (EC) proliferation and migration and the formation of tight adherence junctions. Small GTPases regulate these processes, and their dysregulation may cause vascular pathologies, including atherosclerosis (plaque formation) and angiogenesis. Rho GTPases (Rho, Rac, and Cdc42) play a critical role in vascular pathologies and become activated when they bind to GTP and are inactivated when they bind to GDP. RhoGTPase also plays a role in regulating molecular processes such as contraction, migration, proliferation, and the differentiation of smooth muscle cells and fibroblasts [20,21,22]. This suggests the critical role of RhoGTPase in vessel remodeling, involving the migration and proliferation of ECs and VSMCs. These molecular processes are regulated by various genes and increased or decreased gene expression may alter the migration and proliferation of ECs and VSMCs. Gene expression is regulated by TFs [13] and the inhibition of transcription factor Rho GTPase-activating protein 35 (ARHGAP35), as demonstrated in this study by thrombosed artery RNA seq data, in association with various DEGs involved in VSMC and EC proliferation and migration, angiogenesis, and inflammation (Table 6), suggesting the role of ARHGAP35 in early thrombosis and thereby in AVF maturation failure. However, the underlying mechanistic aspects warrant further investigation.

Hypoxia is another critical factor regulating NIH, EC, and VSMC migration and proliferation, extracellular matrix (ECM) remodeling, and vessel remodeling [23]. Hypoxia is caused due to disruption of the vasa vasorum during the creation of AVF and this induces transcription factors including hypoxia-inducible factors (HIFs) such as HIF-1α, HIF-2α, HIF-3α, HIF-1β (aryl hydrocarbon receptor nuclear translocator; ARNT), and HIF-2β (ARNT2) [23]. In this study, we observed activated ARNT2 in the thrombosed arteries (Table 3) with ApoE, a gene regulating vasodilatation and anti-remodeling, as a target DEG in the data set. This suggests that ARNT2 plays a critical role in AVF non-maturation. Aryl hydrocarbon receptors also play a critical role in ischemia-induced angiogenesis [24] and angiogenesis plays a critical role in plaque vulnerability [25], thrombosis [26], and short expectancy of AVF [27]. Activated ARNT2 in this study might be a therapeutic target to facilitate AVF maturation, as aryl hydrocarbons have been suggested as a therapeutic target in cardiovascular diseases [28,29]. Excessive intimal hyperplasia is also a result of increased ROS production and inflammation, mediated by macrophages that are deficient in MED1 [30]. Inhibited MED1 (Table 6) and the infiltration of pro-inflammatory immune cells, including macrophages, in the thrombosed and intimal hyperplasia region of femoral arteries in this study support the notion of the presence of inflammation [12] and a the possible role of MED1-deficient macrophages in early thrombosis and AVF failure. Inflammation within the evolving plaque is associated with the activation of signal transducers and activators of transcription (STAT)1 and STAT3, and STATs function as intracellular regulators of vascular remodeling [31]. STAT3 is also involved in collagen-induced platelet aggregation and thrombosis [32] and was found to be activated in this study, indicating its role in early arterial thrombosis.

BCL11B (B-cell leukemia 11b) is another transcription factor that was found to be elevated (Table 5) in the upstream network analysis of our data. BCL11B plays a crucial role in the development, proliferation, differentiation, and survival of T cells. Valisno et al. reported the expression of BCL11B in VSMCs and its role in aortic smooth muscle function and vascular stiffness [33]. Since vascular stiffness adversely affects AVF maturation, increased expression of BCL11B in thrombosed arteries suggests its pathological role and its therapeutic potential as a target. Vascular stiffness, mainly arterial stiffness, plays a critical role in hypertension, involving a change in the gene expression and phenotype of VSMCs and ECs and their focal contacts. In ECs and VSMCs, Lin11-Isl1-Mec3 (LIM) domain-containing proteins play a critical role as mechanotransducers [34] and hemodynamic changes occur after AVF creation and contribute to AVF maturation and/or failure. Investigating the role of Isl1, which was found to be activated in this study, will be of significance. Differentiation and proliferation of VSMCs and dysfunction of ECs play a crucial role in stenosis, thrombosis, and remodeling and determine the fate of AVFs. c-Myb, a TF that was found to be activated in this study, regulates VSMC progenitor cells, VSMC differentiation, VSMC proliferation, and arterial remodeling [35,36]. Since early arterial thrombosis, stenosis, and adverse remodeling are associated with early AVF failure in the presence of chronic inflammation [12], considering the mediators regulating arterial remodeling, along with outflow vein remodeling, will be of significance in enhancing vessel and AVF patency.

VSMC proliferation plays a crucial role in the pathogenesis of plaque formation and NIH and cyclin D1 play a critical role [37]. Furthermore, the attenuation of adverse vascular remodeling via CCND1 inhibition supports the therapeutic potential of targeting CCND1 [38]. The increased expression of CCND1 observed in thrombosed vessels (Table 6) in this study indicates that CCND1 might be a therapeutic target to enhance AVF maturation by attenuating NIH and vessel stenosis. The ETS family of transcription factors plays a critical role in plaque formation and vulnerability and increased expression of Ets-1 in VSMCs is associated with plaque vulnerability [39], vascular inflammation, and remodeling [40]. An increased expression of ETS and SPI1 (the transcription factor PU.1) in the thrombosed arteries in this study suggest their role in early thrombosis, mediating early AVF failure. VSMC proliferation and NIH are also regulated by GATA4-mediated cyclin D1 transcription [41] and we observed activated GATA4 in our study (Table 6). Interferon regulatory factor (IRF) 1, which was also found to be activated in this study, is another TF regulating neointima formation after intimal injury and preventing NIH, involving the Ang-II receptor and attenuating vascular remodeling [42]. On the other hand, IRF9, a transcription factor that was found to be activated in this study, is essential for neointima formation and VSMC proliferation after vascular injury [43]. EC proliferation is regulated by another TF, namely, MEF2 (myocyte enhancer factor 2) and we observed activated MEF2C (Table 6) in our study. MEF2 is an upstream regulator of several transcription factors and it promotes an anti-inflammatory and antithrombotic endothelium by regulating TFs, including Klf2, Klf4, and Notch [44]. This suggests the critical role of MEF2C in vessel thrombosis and it might be a therapeutic target for enhancing vessel patency and AVF maturation. This notion is supported by the activated NOTCH4 observed in this study.

**Figure 3:**
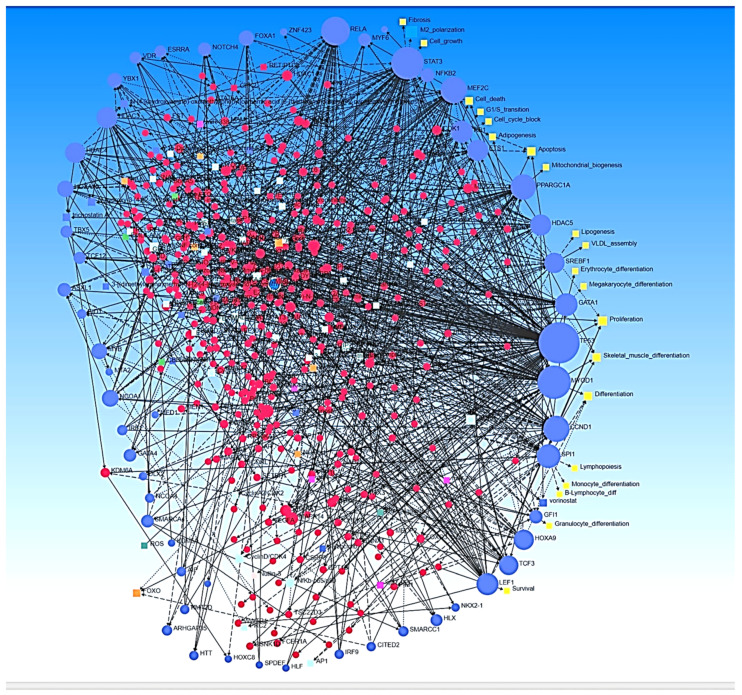
Network analysis with TFs as inputs revealed molecular mechanisms associated with inflammation, hypoxia, adipogenesis, lipogenesis, and mitochondrial biogenesis associated with early thrombosis. Blue circles—input transcription factors, red circles—differentially expressed genes, and yellow squares—molecular mechanisms associated with early thrombosis in arteries involved in arteriovenous fistula.

CITED2 (CBP/p300-interacting transactivator with ED-rich tail 2) suppresses genes mediating angiogenesis and ECM remodeling and regulates the expression of various MMPs [45] and HIF-1α [46] and regulates inflammatory genes involved in fibrosis [47,48]. HIF-1 α, angiogenesis, inflammation, and ECM remodeling play a critical role in AVF maturation and failure, and the regulation of CITED2 expression may play a therapeutic role. The decreased expression of CITED2 observed in this study suggests its probable role in thrombosis and stenosis; however, its role in AVF maturation and failure warrant investigation. ECM remodeling is mediated by various MMPs and the activation of NF-κB regulates the expression of MMPs. The change in hemodynamics associated with shear stress activates NF-κB and regulates long-term flow-induced vascular remodeling [49]. The activated NF-κB2 (nuclear factor Kappa B subunit 2), REL-A (RELA proto-oncogene, NF-κB subunit), and REL-B (RELB proto-oncogene, NF-κB subunit) observed in this study suggest the role of NF-κB in inflammation [12], stenosis, and remodeling, and targeting NF-κB might have therapeutic potential. Network analysis with the transcription factors as inputs revealed molecular mechanisms, including macrophage polarization, the involvement of reactive oxygen species, fibrosis, cell cycle, apoptosis, mitochondrial biogenesis, adipogenesis, and lipogenesis, as well as the interaction of various transcription factors with DEGs (Figure 3). Network analysis also revealed the involvement of FOXO genes and this finding is important because FOXO4 plays a critical role in early inflammatory responses after intimal injury and in apoptosis [50,51].

NIH is a major contributor of arterial restenosis. Vitamin D, exerting its biological effect through the vitamin D receptor (VDR), plays a critical role in mitigating inflammation and excessive NIH [52,53,54]. VDR was inhibited in this study, and this might be due to the presence of chronic inflammation [12]. Decreased expression of VDR is correlated with arterial thrombosis and as per previous reports. The CC chemokine CCL5/RANTES (regulated on activation normal T cell expressed and secreted), which is expressed in VSMCs, regulates NIH, and CCL5 expression in smooth muscle cells is regulated by transcription factor YBX-1, which was found to be activated in this study, coding for Y-box binding protein-1 [55].

Chen et al. [56] reported the role of EHMT2 (also known as G9a) in the regulation of aortic smooth muscle cell death by suppressing autophagy activation independently of proliferation and apoptosis. Histone lysine methyltransferase EHMT2 also plays a role in vasculopathy and vascular inflammation [57]. Another TF, GATA1, regulates EC function and angiogenesis by regulating AGGF1 [58]. Increased expression of HDAC4 is associated with vascular inflammation and associated inflammatory diseases via the activation of autophagy, and knockdown of HDAC4 ameliorates vascular inflammation [59]. We observed HDAC4 to be inhibited in the LR12 + TAK-242-treated arteries and activated in scrambled and ethanol-treated arteries in this study, and this might be the effect of the treatment given to the swine. Similarly, the inhibition of HDAC5 is associated with the reduction of angiotensin II-induced vascular contraction, hypertrophy, and oxidative stress in a mouse model [60], and HDAC5 was found to be inhibited (Table 6) in this study, as an effect of the treatment given. Investigating the role of epigenetic regulators including HDACs and H3K9me3 is of significance because they have abnormal mechanoresponses to arterial laminar shear stress and play a role in EC damage [61], with etiologies playing a critical role in vessel remodeling in AVF.

miRNAs regulate gene expression epigenetically and miR-132 upregulation is associated with reduced VSMC proliferation by attenuating the expression of LRRFIP1 and thus attenuating neointima formation [62]. The transcription factor LRRFIP1 was activated (Table 6), and miR-132 appeared in the data (Supplementary File S1) but was neither activated nor inhibited. The activation of LRRFIP1, a target of miR-132, in thrombosed and stenosed arteries in this study, is indicative of excessive VSMC proliferation. An attenuated expression of mir-133 is associated with the phenotypic switching, proliferation, and migration of VSMCs and thereby plays a role in vascular remodeling [63]. miR-155, by downregulating the soluble guanylyl cyclase (sGC/cGMP) pathway, negatively regulates VSMC functions that are essential for maintaining the VSMC contractile phenotype and vasorelaxation [64]. miR155-5p, which was found to be inhibited in this study, suppresses ACE expression and its downstream production of Ang II, superoxide anions, and inflammatory factors, and it inhibits VSMC migration and oxidative stress in spontaneously hypertensive rats but has no effects on VSMC migration with exogenous Ang II [65]. miR155-5p in adventitial fibroblast-derived extracellular vesicles inhibits the proliferation of VSMCs [66]. Since oxidative stress, hypoxia, and VSMC proliferation play a critical role in AVF maturation/failure, these miRNAs might play an important role. The levels of miR-34a-5p, associated with atherosclerosis and miR-34a-5p, play a crucial role in inflammatory pathology [67] and various studies [68,69] have discussed miR-34a-5p, which was found to be inhibited in this study, as a potential therapeutic target in cardiovascular disease. miR-205-5p, which was found to be activated in this study, suppresses the proliferation of pulmonary VSMCs [70], whereas miR-153, which was found to be inhibited in this study, attenuates hypoxia-induced excessive proliferation and migration of pulmonary arterial SMCs [71]. miR-22, which was found to be inhibited in this study, regulates VSMCs apoptosis (through p38MAPKα) and vascular remodeling in aortic dissection [72], whereas miR-219, which was found to be inhibited in this study, is associated with vascular ischemia during cerebral artery occlusion [73] (Table 1 and Table 2). The expression of these miRNAs in this study and their relationship with vascular pathologies suggest that these miRNAs may be potential therapeutic targets in AVF. Other miRNAs which have been described in relation to vascular remodeling, including miR-1 and miR-204 [74], also appeared in this study but were neither activated nor inhibited. Additionally, this study revealed some novel miRNAs which have not been discussed in the literature (Supplementary File S1) in relation to arterial stenosis and thrombosis involved in AVF and which may cause early AVF maturation failure and thus might have therapeutic potential, since microRNAs play a critical role in AV shunting, angiogenesis, thrombosis, restenosis, NIH, and vessel remodeling [75,76,77,78].

## 5. Future Perspectives

Overall, the results of this study revealed various TFs and microRNAs associated with early arterial thrombosis, stenosis, and nonpatent AVF. The role of various TFs and microRNAs has been discussed in the literature in association with inflammation; the proliferation and migration of VSMCs; the proliferation and dysfunction of ECs; ECM remodeling; and angiogenesis in terms of vascular remodeling, as discussed above. However, the roles of these TFs and microRNAs and others that appeared in this study have not been investigated in relation to AVF maturation and maturation failure. The involvement of other TFs and microRNAs found in this study has been discussed in relation to embryonic organogenesis, vasculogenesis, cardiac remodeling, etc. (Supplementary File S3) this but warrants further research in association with AVF. For example, NRIP1, which was found to be activated in this study, also appeared in another data set [79] in association with blood coagulation, ECM remodeling, inflammatory responses, the TGF signaling pathway, and angiogenic proteins, and all these play a role in AVF maturation and failure. Similarly, TF NUPR1 encoding nuclear protein 1 (Nupr1), a marker for phenotypic modulation (modSMCs) towards a fibroblast-like state, which was found to be activated in this study, also appeared in another single-cell transcriptomic profile of VSMC phenotypes [80]. PPARGC1A, a TF that was observed to be inhibited in this study, also appeared in another genomic and transcriptomic analysis highlighting the role of vascular changes in multiple sclerosis—mainly immune and inflammation-related pathologies mediating perivascular changes [81]. Since perivascular cuffing and perivascular inflammation are associated with arterial thrombosis [12], investigating the role of PPARGC1A will enhance the understanding of the molecular mechanism underlying early AVF failure. SMARCC1 (Swi/snf-related, matrix-associated, actin-dependent regulator of chromatin subfamily c member 1) and SMARCA4 (Swi/snf-related, matrix-associated, actin-dependent regulator of chromatin, subfamily a, member 4) was found to be activated in this study, and Brg1, a central catalytic subunit of the SWI/SNF apparatus, plays a critical role epigenetically in VSMC proliferation [82]. ZNF423 plays a role in activating autophagy by binding to BCAT1 in hypoxic pulmonary artery smooth muscle cells [83], since hypoxia occurs after AVF creation due to disruption of the vasa vasorum. Therefore, ZNF423 might play an important role in AVF maturation and maturation failure. Furthermore, the protein–protein interactions between the genes regulated by these TFs support the notion of their role in early thrombosis, and targeting them might be of therapeutic significance in AVF.

## 6. Conclusions

The results of this study revealed various transcription factors and microRNAs, of which some have implications in vascular remodeling, NIH, VSMC proliferation and migration, and EC proliferation and function (as discussed) and some have been discussed in the literature in other contexts (Supplementary File S3). However, none of these TFs and microRNAs have been investigated in relation to early thrombosis, stenosis, vessel patency, AVF maturation, and maturation failure after AVF creation and thus warrant further research. Since genetic and epigenetic regulation of genes is an evolving research area, investigating genetic and epigenetic mediators playing a critical role in AVF maturation failure and targeting them will be of great significance to improving clinical outcomes.

## Figures and Tables

**Table 1:** MicroRNAs revealed with upstream network analysis: LR12 + TAK242-treated vs. scrambled peptide and vehicle (30% ethanol)-treated arteries. DEGs with higher expression in scrambled peptide-treated group (log_2_ > 2, *p* < 0.05) and with higher expression in LR12 + TAK242-treated group (log_2_ < −2, *p* < 0.05).

Associated with Inhibited Network	DEGs (log_2_ > 2, *p* < 0.05)	DEGs (log_2_ < −2, *p* < 0.05)
mir-133	VCAN	PPARG, TNFSF10
miR-155-5p	BACH1, CD69, CXCL8, PMAIP1, PPL, RIPK1	

**Table 2:** MicroRNAs revealed with causal network analysis: LR12 + TAK242-treated vs. scrambled peptide and vehicle (30% ethanol)-treated arteries. DEGs with higher expression in scrambled peptide-treated group (log_2_ > 2, *p* < 0.05) and with higher expression in LR12 + TAK242-treated group (log_2_ < −2, *p* < 0.05).

Associated with Activated Network	DEGs (log_2_ > 2, *p* < 0.05)	DEGs (log_2_ < −2, *p* < 0.05)	DEGs (log_2_ < 2 or >−2, *p* < 0.05)
miR-205-5p	ADAM8, ADAMTS4, ARG1, CD27, CD3D, CD5, CSF2RB, CXCL8, DNAH11, DUOX2, GP1BA, GP1BB, HPSE, IL1R1, IL2RB, IL7R, KDM6B, LAG3, MMP25, NCOR2, PCDH8, PHACTR1, PPRC1, PTX3, RELT, SEMA4A, SERPINB2, STAT4, STEAP4	ADAMTS8, ADIPOR2, AQP11, BCAM, CAT, CEBPA, CIDEC, DHH, EIF4EBP1, HCAR1, LMO2, MYLPF, PLIN1, PLIN5, PNPLA2, PPARG, RETSAT, SEMA3G, SERPINI1, TEK, WNT11	CD6, CXCR5, EGR2
Associated with inhibited networks			
mir-515	ADAM8, ADAMTS4, ALAS2, BCL3, CCR7, CD27, CD3D, CD69, COL6A3, CXCL8, DUOX2, ECM1, EDIL3, EGR1, FCMR, FERMT3, GP1BA, GPR84, HPSE, ICOS, IL2RB, IL7R, KDM6B, LAG3, MAP3K14, MUC13, MYC, NCOR2, PCDH8, PHACTR1, PLAGL1, PPRC1, PRDM1, PTX3, RCAN1, RNF213, SBNO2, SELP, SEMA4A, SERPINB2, SLAMF7, ST18, STAT4, STC1, TUBB1, VWF	AGT, ALDH9A1, APOD, APOE, ASS1, BCAM, BMX, CAVIN2, CIDEC, CLEC14A, CYP4B1, DDIT4L, ECH1, EIF4EBP1, ENHO, EPHX2, HCAR1, MMD, MYH4, MYL1, OSM, OSMR, PPARG, PLIN5, PNPLA2, PON3, S100A1, SERPINI1, TCAP, TEK, TMOD4, TNNC2, TSPAN5	CD6, CXCR5
mir-153	ADAMTS1, CD5, CD69, CSF2RB, CXCL8, DUOX2, EGR3, FCMR, GP1BA, HAS1, ICOS, IL2RB, ITGA2B, ITGB7, KDM6B, NCOR2, NFATC2, PDE4B, PHACTR1, PRDM1, RNF213, SERPINB2, ST18, STAT4, STEAP4, TEAD3, VDR	AGT, ALDH1A1, APOD, APOE, BCAM, DHH, MYH8, MYL1, MYOT, OSM, OSMR, PLIN1, RAMP2, RETSAT, S100A1, SCD, TCAP, TNNC2, WNT11	
mir-219	ALAS2, BCL3, BLK, CCL22, CSF2RB, CXCL8, DUOX2, FCMR, ICOS, IL2RB, IL7R, ITGA2B, NFATC2, PDE4B, PHACTR1, PLAGL1, PRDM1, PTX3, RNF213, SBNO2, STEAP4, TUBB1, VWF	ALDH1A1, APOD, APOE, ASS1, BCAM, CAT, CAVIN2, LDHB, PPARG, MMD, MYL1, MYLPF, MYOT, OSM, PEX11A, RAMP1, TEK	EGR2, IL9R
mir-22	CCL22, CCR7, DUOX2, HAS1, IL1R1, IL2RB, IL7R, ITGA2B, ITGB7, NFATC2,PTX3, RCAN1, SERPINB2, STEAP4, VDR	ALDH1A2, ASS1, BCAM, MYLPF, TNNC2	EGR2

**Table 3:** Transcription factors revealed with upstream network analysis: LR12 + TAK242-treated vs. contralateral femoral arteries. DEGs with higher expression in LR12 + TAK242-treated group (log_2_ > 2, *p* < 0.05) and with higher expression in contralateral femoral arteries (log_2_ < −2, *p* < 0.05).

Associated with Activated Networks	DEGs (log_2_ > 2, *p* < 0.05)	DEGs (log_2_ < −2, *p* < 0.05)	DEGs (log_2_ < 2 or >−2, *p* < 0.05)
MYOD1	CXCL14, MYH4, MYL1, MYLPF, MYOD1, TNNC2		IGF1
SMARCA4	IL7R, ITGA3, MYL1, MYLPF, TNNC2	CNTN1, MAP1B, MFGE8,	IGF1
MEF2C	MYLPF, MYOD1, MYOT	FRZB, KCNA5,	IGF1
MYF6	EGF, IL15, IL7, MYOD1,		
HOXC8	IL1R2, SLC16A3		
SPDEF	ITGA3	TNC	COL4A2
SPI1	CD1D, CXCL14, IL18, IL1R2, IL7R		CD79B
IRF1	IL15, IL18, IL7, MMP9		
RB1	HOMER1, MYH4, MYH8, MYL1, TCAP, TNNC2		IGF1, COL4A2
ARNT2	APOE, EGF, HOMER1		
SIM1	APOE, EGF, HOMER1		
Associated with inhibited network			
HDAC4	HOMER1, MMP9, MYLK2, MYOT	CNTN1	
KDM5A	HOMER1, MYH4, MYH8, MYL1, TCAP, TNNC2		
HTT	APOE, HOMER1, IL15, MYL1, MYOD1,	PRELP	COL4A2
VDR	IL18, MYH8, S100A8		COL4A2

**Table 4:** Transcription factors (TFs) revealed with causal network analysis: LR12 + TAK242-treated vs. contralateral femoral arteries. DEGs with higher expression in LR12 + TAK242-treated group (log_2_ > 2, *p* < 0.05) and with higher expression in contralateral femoral arteries (log_2_ < −2, *p* < 0.05).

Associated with Activated Networks	DEGs (log_2_ > 2, *p* < 0.05)	DEGs (log_2_ < −2, *p* < 0.05)	DEGs (log_2_ < 2 or >−2, *p* < 0.05)
MYF6	ANK1, APOE, CASQ1, EGF, HOMER1, IL15, IL18, IL7R, MYL1, MYLPF, MYOD1, MYOT, TCAP, TNNC2, TRIM63	AQP5, NOTCH3, TNC	IGF1
MYOD1	ANK1, APOE, CASQ1, EGF, HOMER1, IL18, IL7R, MYL1, MYLPF, MYOT, TCAP, TNNC2, TRIM63	AQP5, NOTCH3, TNC	IGF1
NCOA4	AMPD1, APOE, CD1D, CD247, CD3G, CYGB, DDIT4L, EGF, FGR, HABP2, ICAM3, IL15, IL1R2, IL7R, MMP9, MMRN1, MYH8, MYLPF, MYOT, MYPN, PRKCQ, S100A1, SLN, TFPI, TNNC2, XIRP2	ADAMTSL4, AEBP1, COL8A1, ELN, FBLN5, FRZB, KCNA5, MAP1B, MFGE8, NOTCH3, SLIT3	IGF1, COL4A2
NCOA1	ANK1, CNKSR1, CXCL14, DOK5, EGF, HOMER1, ICAM3, IFIT1, IL15, IL18, IL1R2, IL7, IL7R, MYH4, MYH8, MYL1, MYOT, MYPN, PLIN5, STK17B, TCAP, TNNC2, TRIM63, XIRP2	AEBP1, AQP5, COL8A1, ELN, FBLN5, FMOD, ITGA3, MAP1B, NOTCH3, SLIT3	COL4A2
FOXA1	CD1D, CD247, CNKSR1, CXCL14, CYGB, DDIT4L, FGR, HOMER1, ICAM3, IFIT1, IL15, IL18, IL7, MMP9, MYH4, MYH8, MYL1, MYLPF, MYOD1, MYPN, PLIN5, PRKCQ, S100A1, STK17B, TCAP, TNNC2, XIRP2	ADAMTSL4, BGN, ELN, COL8A1, FBLN5, FMOD, FRZB, ITGA3, MAP1B, TNC, NOTCH3, PRELP, SLIT3	IGF1, COL4A2, CD79B
SMARCC1	CNKSR1, ICAM3, IL7R, MMP9, MYOT	ITGA3, MAP1B, NOTCH3, TNC	IGF1, CD79B COL4A2
SMARCA4	IFIT1, MYL1, MYLPF, TNNC2	CNTN1	IGF1
HOXC8	IL1R2, SLC16A3		
SPDEF		ITGA3, TNC	COL4A2
IRF9	IFIT1, IL18		
Associated with inhibited networks			
AHRR	CD1D, CD247, CD3G, CXCL14, HABP2, ICAM3, IFIT1, IL15, IL18, IL1R2, IL7R, MYH4, MYH8, MYL1, MYLK2, MYOT, MYPN, PLIN5, PRKCQ, STK17B, TCAP, TRIM63, XIRP2	AEBP1, FBLN5, FMOD, FRZB, MFGE8, NOTCH3, SLIT3	COL4A2
HDAC3	ANK1, CXCL14, DOK5, HABP2, IFIT1, IL15, IL18, IL7, IL7R, MYH8, MYLPF, PLIN5, S100A8, SLN, TFPI, TRIM63	AQP5, BGN, COL8A1, ELN, FBLN5, ITGA3, KCNA5, NOTCH3, RASL11B	IGF1, COL4A2
EID1	CNKSR1, CXCL14, CYGB, DDIT4L, DOK5, EGF, FGR, HOMER1, ICAM3, IL15, IL18, IL7, MMP9, MYH4, MYH8, MYL1, MYOT, MYPN, PLIN5, S100A8, STK17B, TMOD4, TNNC2, XIRP2	ADAMTSL4, ADRA1D, AEBP1, AQP5, BGN, FBLN5, FRZB, KCNA5, MAP1B, NOTCH3, SLIT3, TNC	IGF1
HOXA10	APOE, CNKSR1, CXCL14, CYGB, DDIT4L, DOK5, EGF, FGR, HOMER1, ICAM3, IL15, IL18, IL7, MMP9, MYH4, MYH8, MYL1, MYOT, MYPN, PLIN5, S100A8, TK17B, TMOD4, TNNC2, XIRP2	ADAMTSL4, ADRA1D, AEBP1, FBLN5, FRZB, KCNA5, MAP1B, NOTCH3, SLIT3, TNC	CD79B
MTA2	CD1D, COL8A1, CXCL14, DOK5, ICAM3, IFIT1, IL15, IL18, IL1R2, IL7R, MMP9, MYH4, MYH8, MYL1, MYLPF, MYOD1, MYOT, PLIN5, S100A8, SLC16A3, TNNC2, TRIM63	AQP5, ELN, FBLN5, MAP1B,	
HOXA9	ADAMDEC1, C5AR2, CNKSR1, CXCL14, CYGB, DOK5, FGR, ICAM3, IL18, IL7R, MYH4, MYH8, MYL1, MYOD1, MYOT, MYPN, PLIN5, PRKCQ, S100A8, STK17B, TNNC2, TRIM63, XIRP2	ADRA1D, AEBP1, FBLN5, FRZB, ITGA8, KCNA5, MAP1B, NOTCH3, SLIT3, TNC	CD79B
TCF3	CD3G, IL7R, MMP9, MYL1, MYLPF, MYOD1, STK17B, TNNC2	NOTCH3	IGF1, CD79B
AHRR	CD247, CD3G, IL15, IL1R2, IL7R, MYLK2, MYOT, PRKCQ	FBLN5, NOTCH3	COL4A2
AIP	ADRA1D, APOE, CD247, CD3G, IL15, IL1R2, IL7R, PLIN5, PRKCQ, S100A8	FBLN5, NOTCH3	COL4A2
KDM5A	HOMER1, MYH4, MYH8, MYL1, TCAP, TNNC2		
DNMT3L	CASQ1, IFIT1, S100A1, SLIT3, SLN		CD79B
EHF	HOMER1, MMP9, MYH4, MYH8, MYL1, S100A8, TCAP, TFPI, TNC, TNNC2	NOTCH3	IGF1
HDAC4	MYLK2, MYOT		
SOX15	MMP9, MYOD1		

**Table 5:** Transcription factors (TFs) revealed with upstream network analysis: LR12 + TAK242 treated vs. scrambled peptide and vehicle (30% ethanol)-treated arteries. DEGs with higher expression in LR12 + TAK242-treated group (log_2_ < −2, *p* < 0.05) and with higher expression in scrambled peptide-treated group (log_2_ > 2, *p* < 0.05).

Associated with Activated Networks	DEGs (log_2_ < −2, *p* < 0.05)	DEGs (log_2_ > 2, *p* < 0.05)	DEGs (log_2_ < 2 or >−2, *p* < 0.05)
STAT3	AGT, ALDH1A1, CAT, CEBPA, LDHB	ALAS2, ARG1, BCL3, SELP COL5A1, CXCL8, EGR1,EGR3, ICOS, IL1R1,IL2RB, MYC, SBNO2, NFATC2, PLAGL1, PRDM1	CXCR5, EGR2, IL9R
TP53	ALDH1A1, ALDH1A2, APOE, ALDH9A1, ASS1, BMX, CAT, CEBPA, DDIT4L, ECH1, LDHB, PPARG, RAMP2, SCD, TCAP, TMOD4	ADAM8, ARG1, BCL3, CXCL8, EGR1, EGR3, MYC, DMRT1, ECM1, EDIL3, ITGA2B, ITGB7, NCOR2, PDE4B, PRDM1, SELP, SERPINB2, VDR	EGR2
HTT	AGT, APOE, CEBPA, LDHB, MYL1, PPARG	ADAMTS4, BCL3, EGR1, PDE4B, COL6A3, PPRC1, PTPN22, RGS14	CXCR5, EGR2
ETS1	HPSE	CD27, CD69, CRTAM, EGR1, IL2RB, MYC, ITGA2B, PRDM1	
BCL11B		CXCL8	
RELA	AGT, APOE, PPARG	BCL3, CCL22, CCR7, CD69, CXCL8, EGR1, GP1BB, MYC, NFATC2, PDE4B, PRDM1, PTX3, SELP	
VDR	AGT, MYH8, PNPLA2, PPARG	CXCL8, EGR1, MYC, ITGB7, STAT4, VDR	
HDAC4	MYLK2, MYOT	SERPINB2	EGR2
RELB		CXCL8, MYC, PRDM1, STAT4	
KDM5A	MYH4, MYH8, MYL1, TCAP TNNC2		
ASXL1	PLIN1, PPARG, SCD		
LEF1		ECM1, MYC, PRDM1	
NFKB2		CCR7, CXCL8, MYC	CXCR5
NUPR1	MMD	ABL2, CXCL8, MYC	
Associated with inhibited networks			
GFI1	CEBPA,	BCL3, CCR7,CXCL8, IL1R1, ITGB7, MYC, STAT4, VDR	EGR2
PPARGC1A	AGT, APOD, CAT, LDHB, MYC, PLIN5, PNPLA2, SCD, SEMA3G	ADAMTS1, IL1R2, COL6A3, OSMR, STC1	
MYOD1	AGT, ASS1, MYH4, MYL1, MYLPF, TNNC2		
HLX	SEMA3G	CXCL8, EGR1, MYC, PRDM1,	
SPDEF		COL5A1, COL6A3, CXCL8	
RB1	CEBPA, MYH4, MYH8, MYL1, PPARG, RAMP2, TCAP, TNNC2	COL5A1, CXCL8, EGR1, EGR3, MYC, OSMR, PTX3	
IKZF2		CD69, ICOS, IL1R1, LAG3, STAT4	
NCOA1	CEBPA, PPARG	EGR1, MYC	
NKX2-3	CAVIN2	CXCL8, RNF213	
NEUROG1	ASS1, CAVIN2		
KMT2D	IGSF1, PPARG		

**Table 6:** Transcription factors revealed with causal network analysis: LR12 + TAK242-treated vs. scrambled peptide and vehicle (30% ethanol)-treated arteries. DEGs with higher expression in LR12 + TAK242-treated group (log_2_ > 2, *p* < 0.05) and with higher expression in scrambled peptide-treated group (log_2_ < −2, *p* < 0.05).

Associated with Activated Networks	DEGs (log_2_ > 2, *p* < 0.05)	DEGs (log_2_ < −2, *p* < 0.05)	DEGs (log_2_ < 2 or >−2, *p* < 0.05)
MEF2C	ADIPOR2, AGT, AQP11, ASS1, BACH1, BCAM, BST1, CAVIN2, CEBPA, DDIT4L, ECH1, EPHX2, LDHB, MDK, MYL1, MYLPF, MYOT, PEX11A, PLIN5, PNPLA2, RAMP1, RAMP2, RETSAT, SCD, SLC9A3R2, TEK, TMOD4, TNNC2, TSPAN5	ABL2, ALAS2, ARG1, BLK, CD27, CD3D, CHST2, COL5A1, CXCL8, EGR3, DMRT1, ECM1, FCMR, GP1BA, GP1BB, GPR84, IL1R2, IL2RB, IL7R, ITGA2B, ITGB7, LAG3, LMO2, NFATC2, PLIN1, PLAGL1, PPRC1, PRDM1, PTPN22, RNF213, SEMA4A, SELP, ST18, STEAP4, TUBB1, VDR, VWF	EGR2, IL9R
ISL1	AGT, ALDH1A1, ALDH1A2, AQP11, BCAM, CAT, CAVIN2, CEBPA, CIDEC, DHH, EIF4EBP1, EPHX2, KLHL31, LDHB, LGALS12, MMD, MYH4, MYL1, MYOT, PEX11A, PNPLA2, RAMP1, RAMP2, RETSAT, SERPINI1, SFRP5, TCAP, TEK, TNNC2,TSPAN5, WNT11	ADAMTS1, ALAS2, BCL3, CD27, COL5A1, COL6A3, CXCL8, DMRT1, EGR1, EGR3, GPR84, HAS1, HPSE, ICOS, IL1R2, IL1RL1, KDM6B, LAG3, LMO2, MYC, MMP25, NFATC2, OSMR, PCDH8, PRDM1, PLAGL1, PLIN1, SBNO2, SEMA4A, SERPINB2, ST18, STEAP4, TCF7, TEAD3, TUBB1	CXCR5, EGR2, IL9R
EHMT2	ALDH1A1, APOE, ASS1, CAT, CAVIN2, CIDEC, CYP4B1, EPHX2, HCAR1, LDHB, MMD, MYL1, MYLPF, PEX11A, PLIN5, PPARG, SCD, TCAP	COL5A1, COL6A3, EGR1, FCMR, GP1BA, HAS1, IL1R1, IL1RL1, IL2RB, ITGA2B, ITGB7, MUC13, NFATC2, PDE4B, PRDM1, PLIN1, PTX3, SERPINB2, TUBB1, VDR, VWF	EGR2
ASXL1	ADRB1, ALDH1A1, ALDH1A2, ALDH9A1, APOD, APOE, BCAM, BMX, CAT, CAVIN2, CEBPA, CIDEC, CYP4B1, DDIT4L, ECH1, EPHX2, HCAR1, MYLPF, PEX11A, PLIN5, PNPLA2, PPARG, RAMP2, RETSAT, SCD, SEMA3G, SERPINI1, TCAP, TMOD4, TNNC2, WNT11	ABL2, ARG1, ADAM8, ADAMTS1, ADAMTS4, BCL3, CCL22, CCR7, CD3D, CD5, CD27, COL6A3, CXCL8, DUOX2, EGR3, ECM1, EDIL3, GP1BA, GPR84, HAS1, HPSE, IL1RL1, IL2RB, ITGB7, ITGA11, KDM6B, LAG3, LMO2, MMP25, MUC13, NAV1, NFATC2, NCOR2, OSM, OSMR, PHACTR1, PLAGL1, PLIN1, PRDM1, RCAN1, RNF213, SBNO2, SELP, SLAMF7, ST18, STAT4, STC1, STEAP4, TUBB1, VDR	CXCR5, IL9R
BCL11B	AGT, ALDH1A2, ALDH9A1, APOD, APOE, ASS1, BMX, CAVIN2, CIDEC, CLEC14A, CYP4B1, DDIT4L, ECH1, EIF4EBP1, ENHO, EPHX2, HCAR1, HPSE, LDHB, MMD, MYLPF, PEX11A, PPARG, RAMP1, SERPINI1, TCAP, TEK, TMOD4, TSPAN5, WNT11	ADAM8, ADAMTS4, ALAS2, BCL3, CCL22, CCR7, CD3D, CD27, CD69, COL6A3, CXCL8, DUOX2, ECM1, EGR1, FERMT3, GP1BA, GPR84, MYC, EDIL3, ICOS, IL1R2, IL2RB, ITGB7, IL7R, KDM6B, LAG3, MUC13, NCOR2, OSM, OSMR, PHACTR1, PCDH8, PLAGL1, PLIN5, PRDM1, PPRC1, PTX3, RCAN1, RNF213, SBNO2, SEMA4A, SERPINB2, SLAMF7, ST18, STAT4, TCF7, TUBB1,VWF	CD6, CXCR5, EGR2
ANKRD42	AGT, ALDH1A2, ALDH9A1, APOD, APOE, ASS1, BMX, CAVIN2, CIDEC, CLEC14A, CYP4B1, DDIT4L, ECH1, EIF4EBP1, ENHO, EPHX2, HCAR1, HPSE, LDHB, MMD, MYLPF, PEX11A, PPARG, RAMP1, SERPINI1, TCAP, TEK, TMOD4, TSPAN5, WNT11	ADAM8, ADAMTS4, ALAS2, BCL3, CCL22, CCR7, CD3D, CD27, CD69, COL6A3, CXCL8, DUOX2, ECM1, EGR1, FERMT3, ICOS, EDIL3, GP1BA, GPR84, IL1R2, IL2RB, ITGB7, IL7R, KDM6B, LAG3, MUC13, MYC, NCOR2, OSM, OSMR, PCDH8, PHACTR1, PLAGL1, PLIN5, PPRC1, PRDM1, PTX3, RCAN1, RNF213, SBNO2, SEMA4A, SERPINB2, SLAMF7, ST18, STAT4, TCF7, TUBB1, VWF	CD6, CXCR5, EGR2
YBX1	AGT, ALDH1A2, ALDH9A1, APOD, APOE, ASS1, BMX, CAVIN2, CIDEC, CLEC14A, CYP4B1, DDIT4L, ECH1, EIF4EBP1, ENHO, EPHX2, HCAR1, HPSE, LDHB, MMD, MYLPF, PEX11A, PPARG, RAMP1, SERPINI1, TCAP, TEK, TMOD4, TSPAN5, WNT11	ADAM8, ADAMTS4, ALAS2, BCL3, CCL22, CCR7, CD3D, CD27, CD69, COL6A3, CXCL8, DUOX2, EGR1, FERMT3, GP1BA, GPR84, ICOS, MYC, ECM1, EDIL3, IL1R2, IL2RB, ITGB7, IL7R, KDM6B, LAG3, MUC13, NCOR2, OSM, OSMR, PCDH8, PHACTR1, PRDM1, PPRC1, PLAGL1, PLIN5, PTX3, RCAN1, RNF213, SBNO2, SEMA4A, SERPINB2, SLAMF7, ST18, STAT4, TCF7, TUBB1, VWF	CD6, CXCR5, EGR2
GATA4	AGT, APOE, ASS1, CAT, CEBPA, CLEC14A, EIF4EBP1, MYL1, MYLPF, MYOT, PPARG, RAMP1, TCAP	ADAMTS4, ALAS2, BCL3, BLK, CCL22,CCR7, CD69, CSF2RB, CXCL8, DMRT1, FERMT3, GP1BA, HAS1, ICOS, IL1R1, IL1R2, IL1RL1, IL7R, ITGA2B, KDM6B, LMO2, MYC, NFATC2, OSM, OSMR, PDE4B, PLAGL1, PRDM1, PTX3, RCAN1, SELP, SERPINB2, ST18, TUBB1, VDR	CXCR5
CCND1	AGT, APOE, BCAM, CAT, CIDEC, CYP4B1, DHH, HCAR1, MMD, MYH4, MYH8, MYL1, MYLPF, MYOT, PEX11A, PLIN5, PNPLA2, PPARG, RAMP2, RBP7, SCD, TCAP, TNNC2, TSPAN5, WNT11	ALAS2, ARG1, CCL22, CCR7, CD5, CD27, COL5A1, EGR3, GP1BA, GP1BB, HAS1, ICOS, IL2RB, ITGA2B, IL7R, LMO2, MYC, MAP3K14, MUC13, NCOR2, NFATC2, PCDH8, PLAGL1, PLIN1, PPRC1, PTX3, SBNO2, STEAP4, TEAD3, TUBB1, VDR	CD6, IL9R
MYB	AGT, APOE, BCAM, CAVIN2, MMD, RCAN1	ALAS2, ARG1, BCL3, CCL22, CCR7, CD69, CXCL8, EGR1, FCMR, GP1BA, HPSE, ICOS, IL1R1, IL1RL1, IL2RB, IL7R, KDM6B, LMO2, MYC, ITGA2B, PDE4B, PRDM1, PTPN22, PTX3, SERPINB2, ST18, STEAP4, TUBB1, VDR, VWF	EGR2
LRRFIP1	AGT, ALDH1A1, ALDH1A2, APOE, CEBPA, PPARG, WNT11	BCL3, CCL22, CCR7, CD69, CXCL8, EGR1, HAS1, ICOS, IL7R, KDM6B, MYC, ITGA2B, PDE4B, PLIN1, PRDM1, PTX3, SERPINB2, ST18, STAT4, TCF7	EGR2
ESRRA	AGT, ALDH1A1, ALDH1A2, APOD, CAT, CEBPA, EGFL7, LDHB, MYH8, PLIN5, PNPLA2, PPARG, SEMA3G, STC1, WNT11	ADAM8, ADAMTS1, CCL22, CCR7, CXCL8, COL6A3, EGR3, IL1R1, IL1R2, IL2RB, MYC, OSMR, PLIN1, PDE4B, PRDM1, PTX3, STEAP4, TCF7	
NOTCH4	AGT, CEBPA, LDHB, MYL1, PPARG, SCD, TCAP, TEK	ALAS2, BCL3, CCL22, CCR7, CD27, CD69, CXCL8, EGR1, ICOS, IL7R, IL2RB, KDM6B, LMO2, OSM, PRDM1, PTX3, SERPINB2, STAT4, ST18, VWF	
GATA1	AGT, ALDH1A1, CAT, CAVIN2, LDHB, MMD, PPARG	ALAS2, BCL3, CCL22, CXCL8, CD69, DUOX2, FCMR, GP1BA, GPR84, IL7R, ICOS, IL1R1, IL1R2, IL1RL1, ITGA2B, LMO2, MYC, NFATC2, PHACTR1, PLAGL1, PDE4B, PTPN22, RNF213, SBNO2, STAT4, ST18, TUBB1, VDR, VWF	IL9R
TBX5	AGT, APOE, ASS1, MYL1, MYLPF, MYOT, PPARG	BCL3, BLK, CCL22, CCR7, IL7R, KDM6B, CD69, CXCL8, DMRT1, EGR1, ICOS, MYC, NFATC2, PLAGL1, PTX3, SERPINB2, ST18, STAT4	EGR2
NRIP1	AGT, APOD, BCAM, CAT, CYP4B1, LDHB, PLIN5, PNPLA2, PPARG, SCD, SEMA3G, STC1	ADAMTS1, ARG1, COL6A3, IL7R, CXCL8, EGR1, IL1R1, IL2RB, OSMR, PLIN1, PTX3, STEAP4	EGR2
TRIM32	AGT, APOE, CEBPA, PPARG	ARG1, BCL3, CCL22, CCR7, CD69, EGR1, ICOS, IL7R, KDM6B, PRDM1, PTX3, SELP, SERPINB2, STAT4, ST18	CXCR5
BCL11B		CXCL8, IL7R	
HDAC4	MYLK2, MYOT		EGR2
Associated with inhibited networks			
HDAC5	AGT, ALDH1A1, ALDH1A2, APOE, ASS1, CASQ1, CAT, CEBPA, CIDEC, CYP4B1, DDIT4L, ECH1, EIF4EBP1, EPHX2, HCAR1, LDHB, MDK, MMD, MYC, MYH4, MYH8, MYL1, MYLPF, MYOT, PEX11A, PLIN5, PNPLA2, PPARG, RAMP2, RETSAT, SCD, SERPINI1, TEK, TNNC2	ALAS2, ARG1, BLK, CD3D, CD27, CHST2, COL5A1, CSF2RB, DUOX2, EGR3, FCMR, GP1BA, GP1BB, IL1R2, IL1RL1, DMRT1, ITGA2B, KDM6B, LMO2, MUC13, NFATC2, OSM, OSMR, PHACTR1, PLAGL1, PLIN1, PTPN22, RCAN1, RNF213, SBNO2, SELP, STC1, ST18, STAT4, TUBB1, VDR	CD6, EGR2, IL9R
SREBF1	ADRB1, ALDH1A1, ALDH1A2, ALDH9A1, APOD, APOE, BCAM, BMX, CAT, CAVIN2, CEBPA, CIDEC, CYP4B1, DDIT4L, ECH1, EPHX2, HCAR1, MDK, MYL1, MYLPF, PEX11A, PLIN5, PNPLA2, PPARG, RAMP2, RETSAT, SCD, SEMA3G, SERPINI1, TCAP, TMOD4, TNNC2, WNT11	ABL2, ADAMTS1, ADAM8, ADAMTS4, ARG1, BCL3, CCL22, CCR7, CD3D, CD5, CD27, COL6A3, DUOX2, EGR3, ECM1, EDIL3, GP1BA, GPR84, HAS1, HPSE, IL1R2, IL1RL1, ITGA11, ITGB7, KDM6B, LAG3, LMO2, MMP25, MUC13, MYC, NAV1, NFATC2, OSM, OSMR, PHACTR1, NCOR2, PLAGL1, PLIN1, PRDM1, RCAN1, RNF213, SBNO2, SELP, SLAMF7, STC1, ST18, STAT4, TUBB1, VDR	CXCR5, IL9R
MED24	ADRB1, AGT, ALDH1A2, ALDH9A1, APOD, APOE, BMX, CAT, CAVIN2, CEBPA, CIDEC, CYP4B1, DDIT4L, DHH, ECH1, EPHX2, HCAR1, MYH4, MYH8, MYLPF, MYOT, PEX11A, PLIN5, PNPLA2, PPARG, RAMP2, RETSAT, SCD, SEMA3G, SERPINI1, SGK2, STC1, TCAP, TMOD4, TNNC2, WNT11	ABL2, ADAM8, ADAMTS4, ADIPOR2, ARG1, BCL3, CCL22, CCR7, CD3D, CD5, CD27, CXCL8, DUOX2, EGR1, EGR3, HAS1, HPSE, IL1RL1, ECM1, EDIL3, GP1BA, GPR84, ITGB7, ITGA11, KDM6B, LAG3, LMO2, MAP3K14, MUC13, MYC, MMP25, NCOR2, NFATC2, NAV1, OSM, PHACTR1, PLAGL1, PLIN1, PRDM1, RCAN1, RNF213, SBNO2, SEMA4A, SELP, ST18, STAT4, SLAMF7, TEAD3, TUBB1, VDR	CXCR5, IL9R
Ncoa6	ADRB1, ALDH1A1, ALDH1A2, ALDH9A1, APOD, APOE, BCAM, BMX, CAT, CAVIN2, CEBPA, CIDEC, CYP4B1, DDIT4L, ECH1, EPHX2, HCAR1, MYLPF, PEX11A, PLIN5, PNPLA2, RAMP2, RETSAT, SCD, SEMA3G, SERPINI1, TCAP, TMOD4, TNNC2, WNT11	ABL2, ADAMTS1, ADAMTS4, ADAM8, ARG1, BCL3, CCL22, CCR7, CD3D, CD5, CD27, COL6A3, CXCL8, DUOX2, EGR3, EGR1, GP1BA, GPR84, IL1RL1, IL2RB, ECM1, EDIL3, HAS1, HPSE, ITGB7, ITGA11, KDM6B, LAG3, LMO2, MMP25, MUC13, NAV1, NCOR2, NFATC2, OSM, OSMR, PHACTR1, PLAGL1, PLIN1, PRDM1, RCAN1, RNF213, SBNO2, SELP, SLAMF7, STC1, ST18, STEAP4, STAT4, TUBB1, VDR	CXCR5, IL9R
ZBTB32	AGT, ALDH1A2, ALDH9A1, APOD, APOE, ASS1, BMX, CAVIN2, CIDEC, CLEC14A, CYP4B1, DDIT4L, ECH1, EIF4EBP1, ENHO, EPHX2, HCAR1, LDHB, MMD, MYLPF, PEX11A, PPARG, RAMP1, SERPINI1, TCAP, TEK, TMOD4, TSPAN5, WNT11	ADAM8, ADAMTS4, ALAS2, BCL3, CCL22, CCR7, CD3D, CD27, CD69, COL6A3, CSF2RB, CXCL8, DUOX2, ECM1, EDIL3, FERMT3, GP1BA, GPR84, HPSE, ITGB7, ICOS, IL1R2, IL2RB, IL7R, KDM6B, LAG3, MYC, MUC13, NCOR2, OSM, OSMR, PHACTR1, PCDH8, PLAGL1, PLIN5, PPRC1, PRDM1, PTX3, RCAN1, RNF213, SBNO2, SEMA4A, SERPINB2, SLAMF7, ST18, STAT4, TCF7, TUBB1, VWF	CD6, CXCR5, EGR2
NKX2-1	AGT, ALDH1A2, APOE, ASS1, BACH1, CAT, CAVIN2, CEBPA, DHH, EPHX2, LDHB, LGALS12, MDK, MMD, MYH4, MYH8, MYL1, MYLPF, PEX11A, RAMP1, SCD, TCAP, WNT11	ADAM8, ALAS2, CD3D, COL5A1, COL6A3, CSF2RB, EGR3, FCMR, HAS1, ICOS, IL1R2, IL1RL1, IL2RB, IL7R, LAG3, MYC, ITGA2B, MMP25, NAV1, NCOR2, NFATC2, OSM, OSMR, PLAGL1, PLIN1, PRDM1, PTX3, RCAN1, SBNO2, SELP, SERPINB2, TCF7, TUBB1, VDR, VWF	CD6, CXCR5, IL9R
NEUROG2	ADRB1, AGT, ALDH1A1, ALDH1A2, ALDH9A1, APOD, ASS1, BACH1, BCAM, BMX, CAVIN2, CIDEC, CYP4B1, DDIT4L, DHH, ECH1, EIF4EBP1, EPHX2, HCAR1, LDHB, MDK, MMD, MYH4, MYL1, MYLPF, PLIN5, RAMP2, TCAP, TEK, TMOD4, WNT11	ARG1, BCL3, CCL22, CCR7, COL5A1, EGR1, EGR3, HAS1, HPSE, IL1R1, IL2RB, ECM1, EDIL3, FCMR, GP1BA, ITGB7, KDM6B, MMP25, MUC13, NFATC2, OSM, OSMR, PLAGL1, PLIN1, RCAN1, SBNO2, ST18, STAT4, STEAP4, TCF7, TUBB1, VWF	CXCR5, EGR2, IL9R
DLX2	ALDH1A2, APOE, AQP11, ASS1, BACH1, BST1, CEBPA, EIF4EBP1, ENHO, FFAR4, MMD, TSPAN5, WNT11	ADAM8, ARG1, BCL3, CD69, CD3D, CSF2RB, CXCL8, DUOX2, EGR1, EGR3, FCMR, GPR84, HSH2D, IL1R2, IL1RL1, IL2RB, ITGB7, KDM6B, LAG3, MYC, NFATC2, OSM, MMP25, NAV1, PHACTR1, PRDM1, PTX3, RNF213, SBNO2, SELP. SEMA4A, SLAMF7, SERPINB2, ST18, STAT4, TCF7, VDR	EGR2
NCOA6	ADRB1, AGT, APOD, APOE, AQP11, ASS1, CAVIN2, CEBPA, DNAH11, EIF4EBP1, ENHO, EPHX2, HCAR1, MMD, MYH4, MYLPF, PEX11A, PON3, PPARG, RETSAT, S100A1, SEMA3B, SERPINI1, SLC9A3R2, TNNC2, WNT11	ADAMTS1, ADAMTS4, ADIPOR2, CD27, CD69, CD3D, CD5, COL5A1, CSF2RB, COL6A3, CXCL8, EGR1, FCMR, GP1BA, HAS1, ICOS, IL1R2, IL1RL1, ITGA2B, KDM6B, LMO2, MAP3K14, MYC, OSM, PHACTR1, PCDH8, PLAGL1, PLIN1, PTX3, RELT, RNF213, SBNO2, SEMA4A, STAT4, STC1, TCF7, TUBB1, VWF, VDR	IL9R
FOXJ1	ALDH1A2, APOE, AQP11, ASS1, BACH1, BST1, CEBPA, EIF4EBP1, ENHO, FFAR4, MMD, TSPAN5, WNT11	ADAM8, ARG1, BCL3, CD3D, CD69, CSF2RB, CXCL8, DUOX2, EGR1, EGR3, FCMR, GPR84, HSH2D, IL1R2, IL1RL1, IL2RB, ITGB7, KDM6B, LAG3, MMP25, NAV1, NFATC2, OSM, PHACTR1, PRDM1, PTX3, RNF213, SBNO2, SELP, SEMA4A, SLAMF7, SERPINB2, ST18, STAT4, TCF7, VDR	EGR2
FOXD1	ALDH1A2, APOE, AQP11, ASS1, BACH1, BST1, CEBPA, ENHO, FFAR4, MMD, TSPAN5, WNT11	ADAM8, ARG1, BCL3, CD3D, CD69, CSF2RB, CXCL8, DUOX2, EGR1, EGR3, FCMR, GPR84, HSH2D, ITGB7, IL1R2, IL1RL1, IL2RB, KDM6B, LAG3, MYC, MMP25, NAV1, PRDM1, PTX3, NFATC2, OSM, PHACTR1, RNF213, SBNO2, SELP, SERPINB2, SEMA4A, SLAMF7, ST18, STAT4, TCF7, VDR	EGR2
ARHGAP35	ADRB1, AGT, ALDH1A1, ALDH9A1, APOD, ASS1, BACH1, BCAM, BMX, CIDEC, CYP4B1, DDIT4L, DHH, ECH1, EIF4EBP1, HCAR1, KDM6B, LDHB, MMD, MYH4, MYLPF, PEX11A, PLIN5, RAMP1, RAMP2, SCD, TCAP, TEK, TMOD4, TNNC2	ALAS2, BCL3, COL5A1, CSF2RB, CXCL8, DUOX2, EGR3, FCMR, GP1BA, IL1R1, IL1RL1, IL2RB, ECM1, EDIL3, ITGB7, MUC13, NFATC2, OSM, OSMR, PLAGL1, PLIN1, PTX3, RCAN1, SBNO2, STEAP4, ST18, STAT4, TUBB1, VWF	CXCR5, IL9R
MED1	ADRB1, ALDH1A2, APOE, CAT, CAVIN2, CEBPA, CIDEC, CYP4B1, HCAR1, MUC13, PEX11A, PLIN5, PNPLA2, PPARG, SCD, SEMA3B	ARG1, CCL22, CCR7, CD69, COL5A1, COL6A3, EGR1, IL1R2, IL1RL1, IL2RB, MYC, PDE4B, PLIN1, PRDM1, STC1, VDR	
Ncoa6	ALDH1A2, APOE, CAT, CAVIN2, CEBPA, CIDEC, CYP4B1, HCAR1, MUC13, PEX11A, PLIN5, SCD	ARG1, CCL22, CCR7, CXCL8, PLIN1, PRDM1, VDR	
ZNF423	ALDH1A2, APOE, CAT, CAVIN2, CEBPA, CIDEC, CYP4B1, HCAR1, MUC13, PEX11A, PLIN5, PPARG, SCD	ARG1, CCL22, CCR7, CXCL8, MYC, PLIN1, PRDM1, VDR	
CITED2	AGT, ALDH1A2, APOE, CAT, CAVIN2, CIDEC, CYP4B1, HCAR1, PEX11A, PLIN5, PPARG, SCD, SEMA3B, TEK	ALAS2, ARG1, BCL3, CCL22, CCR7, CD69, CXCL8, EGR1, EGR3, ICOS, IL7R, KDM6B, MUC13, MYC, PLIN1, PTX3, SERPINB2, ST18, STAT4, VDR	
CBFA2T3	AGT, ALDH1A1, CAT, CAVIN2, LDHB, MMD, PPARG	ALAS2, BCL3, CD69, CXCL8, CCL22, DUOX2, FCMR, GP1BA, GPR84, IL7R, ICOS, IL1R1, IL1R2, IL1RL1, ITGA2B, LMO2, MYC, NFATC2, PHACTR1, PLAGL1, PDE4B, PTPN22, RCAN1, RNF213,SBNO2, STAT4, ST18, TUBB1, VDR, VWF	IL9R
TCF12	AGT, ASS1, EIF4EBP1, MYL1, MYLPF, PPARG, TNNC2	CD3D, CD5, CXCL8, ICOS, IL1RL1, IL2RB, ITGB7, IL7R, LMO2, PRDM1, SEMA4A, SLAMF7	IL9R
HLF	APOE, ASS1, CAT, CEBPA, CIDEC, ECH1, MMD, PEX11A, PLIN5, PNPLA2, PPARG, RETSAT, SCD	ARG1, LMO2, MYC, PLIN1,	
IKZF2		CD69, ICOS, IL1R1, LAG3, STAT4	
MYOD1	AGT, ASS1, MYL1, MYLPF, TNNC2		
GFI1	CEBPA	BCL3, CCR7, IL1R1, IL7R, ITGB7, STAT4	
HLX	RAMP1, RAMP2, SEMA3G, TSPAN5	BCL3, CXCL8, EGR1, KDM6B, MYC, PRDM1	
SPDEF		COL5A1, COL6A3, CXCL8	

## Data Availability

All data supporting the results of this manuscript have been included in this manuscript along with Supplementary Files. Bulk RNA seq (Fastq. Files) can be provided from the corresponding author on request. The data have been uploaded at NCBI SRA (https://www.ncbi.nlm.nih.gov/sra (accessed on 26 April 2022)) with submission # SUB11379495.

## Supplementary Materials

The following supporting information can be downloaded at: https://www.mdpi.com/article/10.3390/proteomes10020014/s1, Supplementary File S1: List of all microRNAs; Supplementary File S2: List of all transcription factors; Supplementary File S3: Details of transcription factors.
